# Assembly of transgenic human P301S Tau is necessary for neurodegeneration in murine spinal cord

**DOI:** 10.1186/s40478-019-0695-5

**Published:** 2019-03-18

**Authors:** Jennifer A. Macdonald, Iraad F. Bronner, Lesley Drynan, Juan Fan, Annabelle Curry, Graham Fraser, Isabelle Lavenir, Michel Goedert

**Affiliations:** 1 MRC Laboratory of Molecular Biology, Francis Crick Avenue, Cambridge, CB2 0QH UK; 2 Wellcome Sanger Institute, Wellcome Genome Campus, Hinxton, CB10 1SA UK; 3 AstraZeneca, Sir Aaron Klug Building, Granta Park, Cambridge, CB21 6GH UK; 4 Kymab, The Bennet Building, Babraham Research Campus, Cambridge, CB22 3AT UK

**Keywords:** Tau assembly, Neurodegeneration, Tauopathy, β-Sheet structure, Tau filaments, Sarkosyl-insoluble tau

## Abstract

A pathological pathway leading from soluble monomeric to insoluble filamentous Tau is characteristic of many human neurodegenerative diseases, which also exhibit dysfunction and death of brain cells. However, it is unknown how the assembly of Tau into filaments relates to cell loss. To study this, we first used a mouse line transgenic for full-length human mutant P301S Tau to investigate the temporal relationship between Tau assembly into filaments, assessed using anti-Tau antibody AT100, and motor neuron numbers, in the lumbar spinal cord. AT100 immunoreactivity preceded nerve cell loss. Murine Tau did not contribute significantly to either Tau aggregation or neurodegeneration. To further study the relevance of filament formation for neurodegeneration, we deleted hexapeptides ^275^VQIINK^280^ and ^306^VQIVYK^311^, either singly or in combination, from human 0N4R Tau with the P301S mutation. These hexapeptides are essential for the assembly of Tau into filaments. Homozygous mice transgenic for P301S Tau with the hexapeptide deletions, which expressed Tau at a similar level to the heterozygous line transgenic for P301S Tau, had a normal lifespan, unlike mice from the P301S Tau line. The latter had significant levels of sarkosyl-insoluble Tau in brain and spinal cord, and exhibited neurodegeneration. Mice transgenic for P301S Tau with the hexapeptide deletions failed to show significant levels of sarkosyl-insoluble Tau or neurodegeneration. Recombinant P301S Tau with the hexapeptide deletions failed to form β-sheet structure and filaments following incubation with heparin. Taken together, we conclude that β-sheet assembly of human P301S Tau is necessary for neurodegeneration in transgenic mice.

## Introduction

The assembly of Tau protein into abnormal filaments characterises many human neurodegenerative diseases [16]. Assemblies appear to spread through specific neuronal networks in each disease, with short filaments having the greatest seeding activity [22]. Six Tau isoforms are expressed in normal adult human brain – three isoforms with four microtubule-binding repeats each (R1, R2, R3, R4; 4R Tau) and three isoforms lacking R2 (3R Tau) [19]. Each repeat is 31 or 32 amino acids in length. Tau filaments can be composed of either 3R or 4R Tau, or of 3R + 4R Tau. High-resolution structures of human brain Tau filaments made of 3R Tau and of 3R + 4R Tau established the existence of molecular conformers of aggregated Tau [11, 14]. The identification of disease-causing mutations in *MAPT*, the Tau gene, in cases of frontotemporal dementia, showed that dysfunction of Tau protein is sufficient to cause neurodegeneration and dementia [21, 35, 40].

In humans, mutation P301S in R2 of Tau gives rise to an early-onset form of frontotemporal dementia [5]. We produced and characterised a transgenic mouse line, which expresses full-length human 0N4R P301S Tau, under the control of the murine Thy1 promoter [1]. It exhibits the essential characteristics of human Tauopathies, including Tau hyperphosphorylation, abundant Tau filaments in nerve cells and neurodegeneration. Even though the propagation of Tau pathology correlates best with the presence of short Tau filaments [22], the connection between Tau assembly and neurodegeneration is only incompletely understood. Nerve cell loss in the absence of abundant Tau filaments has been reported in the rTg4510 transgenic mouse line, which expresses high levels of human mutant P301L Tau [37, 41]. A subsequent study showed that memory loss in this line correlated with the presence of soluble Tau oligomers [4].

To examine the temporal relationship between assembly and neurodegeneration, we quantitated Tau assembly and nerve cell loss in the ventral horn of the lumbar spinal cord of mice transgenic for human mutant P301S Tau. We assessed Tau assembly using antibody AT100 [27, 50], immunoreactivity of which is closely associated with Tau filaments rich in β-sheets in adult mouse brain [1, 7]. We counted motor neuron numbers using unbiased stereology. We report that AT100 immunoreactivity preceded nerve cell loss, with murine Tau playing no significant part.

Two hexapeptides (residues ^275^VQIINK^280^ in R2 and ^306^VQIVYK^311^ in R3) are important for Tau filament formation [25, 38, 39, 45, 46]. The local structure encompassing residues 306–311 regulates Tau assembly, and mutations P301L and P301S increase susceptibility to conformational changes that expose this hexapeptide motif [28]. To further examine the relationship between β-sheet-dependent assembly of Tau and neurodegeneration, we generated homozygous mice transgenic for human P301S Tau that lacked residues ^275^VQIINK^280^, ^306^VQIVYK^311^ or both hexapeptides and expressed levels of human mutant Tau that were similar to those of the heterozygous line transgenic for full-length P301S Tau. While mice transgenic for full-length P301S Tau had a heavy load of sarkosyl-insoluble Tau in spinal cord at 16 months of age and suffered from a severe paraparesis, the deletion lines did not develop significant levels of sarkosyl-insoluble Tau and had a normal lifespan. In addition, recombinant P301S Tau with these hexapeptide deletions failed to form β-sheet structure and filaments following incubation with heparin.

## Materials and methods

### Transgenic mice

Homozygous human 0N4R P301S Tau transgenic mice [1] on a pure C57BL/6 JAX background and age-matched C57BL/6 JAX mice were analysed at postnatal day 20, monthly from 1 to 6 months and at 7–8 months (end stage), with 5 animals per group. The minimum number of animals needed was determined by power analysis (GraphPad StatMate) with effect size = 20%, significance level = 0.05, power = 90%. End stage was defined as the presence of a severe paraparesis involving one or both hindlimbs. We also analysed homozygous P301S Tau transgenic mice on a murine Tau null background at end stage (*n* = 5). This line was created by crossing mice transgenic for human mutant P301S Tau with a murine Tau knockout line [43]. The latter is knockout for most of mouse Tau, but expresses its first 31 amino acids. Following back-crossing with a pure C57BL/6 JAX line for six generations, MAX-BAX analysis (Charles River) showed that the human P301S Tau x mouse Tau knockout line was on a pure C57BL/6 JAX background. Three new transgenic mouse lines expressing 0N4R P301S Tau with either deletions of ^275^VQIINK^280^ (line Δ1), ^306^VQIVYK^311^ (line Δ2) or both (^275^VQIINK^280^ and ^306^VQIVYK^311^) (line Δ3) were produced. Site-directed mutagenesis (QuikChange, Agilent Technologies, UK) was used to introduce the hexapeptide deletions into the 0N4R P301S Tau cDNA. Mutated cDNAs were subcloned into pCR2.1 TOPO vectors and a Kozak consensus sequence was introduced upstream of the start codon, followed by subcloning into the XhoI site of the murine Thy1.2 genomic expression vector. Transgenic mice were produced using pronuclear injection of (C57BL/6J x CBA/CA) F1 embryos. Founders were identified by polymerase chain reaction (PCR) of lysates from tail biopsies using 5′- CACAGACACACACCCAGGACATAG-3′ (forward primer) and 5′-CCACCTCCTGGTTTATGATGGATG-3′ (reverse primer). Homozygosity was determined by quantitative PCR and confirmed by mating with wild-type mice.

### ELISA

Brains and spinal cords were homogenised in 2 ml/g buffer [25 mM Tris-HCl, pH 7.4, 150 mM NaCl, 1 mM EDTA, 1 mM EGTA, 5 mM sodium pyrophosphate, 10 mM β-glycerophosphate, 30 mM sodium fluoride, 10 mM sodium vanadate, 10 mM PMSF and one tablet of complete protease inhibitor cocktail (Roche) per 20 ml buffer], followed by a 30 min centrifugation at 150,000 g. The supernatants from at least three mice per group were diluted 1:150 (30–60 ng protein/ml) in reagent diluent (R&D Systems) and incubated overnight at 4 °C in 96-well ELISA plates, which were blocked with phosphate-buffered saline (PBS) containing 0.2% Tween-20 and 3% bovine serum albumin (BSA) for 1 h at 37 °C. To assess human Tau transgene expression levels, the supernatants were then incubated with human-specific [27] anti-Tau antibody HT7 (1:6,000, Fisher Scientific) for 2 h, followed by horseradish peroxidase-conjugated anti-mouse antibody (1:50,000, Bio-Rad) for 1 h. Following a colorimetric reaction, the plates were read on a Tecan plate reader. Purified recombinant Tau and brain lysates of heterozygous mice transgenic for human mutant P301S Tau were used as standards.

### Sarkosyl extraction

The above pellets were resuspended in 10 mM Tris-HCl, pH 7.4, 800 mM NaCl, 1 mM EGTA, 10 mM PMSF, 10% sucrose and one tablet/20 ml buffer of complete protease inhibitor cocktail (Roche). After a 20 min centrifugation at 6,000 g, the supernatants were incubated with 1% sarkosyl (Sigma) for 90 min at room temperature, followed by a 1 h centrifugation at 150,000 g. The sarkosyl-insoluble pellets were resuspended in 150 μl/g tissue 50 mM Tris-HCl, pH 7.4 and stored at 4 °C for immunoblotting.

### Immunoblotting

Samples were run on Novex 8% or 10% Tris-Glycine gels (ThermoFisher Scientific) and transferred onto nitrocellulose membranes. The blots were incubated overnight with primary antibodies, followed by either anti-mouse or anti-rabbit HRP conjugated secondary antibodies, and the signal was visualised by enhanced chemiluminescence (GE Healthcare).

### Antibodies

To detect assembled Tau, we used antibody AT100 (ThermoFisher Scientific), which recognises Tau phosphorylated at T212, S214 and T217 [50], and is a marker of filamentous Tau in transgenic mouse brain [1, 7]. To quantify motor neuron loss, we used an anti-NeuN antibody (Millipore). AT100 and anti-NeuN were used at 1:500. For immunoblotting, AT100 was used at 1:1,000. T49 (Merck Millipore) [23] was used at 1:50,000 for detection of murine Tau. HT7 (ThermoFisher Scientific) was used at 1:1000 for detection of human Tau [27].

### Immunohistochemistry

Mice were perfused transcardially with 4% paraformaldehyde in 0.1 M PBS, pH 7.4. Spinal cords were dissected and post-fixed overnight at 4 °C, followed by cryo-protection in 20% sucrose in PBS for a minimum of 24 h. Serial transverse sections (30 μm) of lumbar spinal cord (L3-L5) were cut on a Leica SM2400 microtome (Leica Microsystems) and stored at 4 °C in PBS containing 0.1% sodium azide. Every twelfth section was processed for immunohistochemistry. Endogenous peroxidase activity was quenched by incubation in 0.3% H_2_O_2_ for 30 min. Following a brief wash in PBS + 0.1% Triton X-100 (PBST), the sections were incubated in blocking buffer (PBST + 5% normal horse serum) for 1 h. This was followed by an overnight incubation at 4 °C with primary antibody in blocking buffer. After three rinses with PBST, the sections were incubated with a biotin-conjugated anti-mouse antibody for 1 h at room temperature. Following a further three rinses with PBST, the avidin-biotin-conjugated complex was applied for 1 h. The antigen was visualised with the Vector VIP substrate kit (Vector Laboratories). Tissue sections were mounted on frosted end glass slides (ThermoScientific) and coverslipped.

### Tau expression, purification and assembly

Following subcloning into pRK172, expression and purification of recombinant 0N4R P301S Tau, 0N4R P301S Tau lacking residues ^275^VQIINK^280^ (Δ1), ^306^VQIVYK^311^ (Δ2), or both hexapeptides (Δ3) were done as described [10, 18]. Tau concentrations were determined by amino acid analysis. For filament assembly, Tau proteins (135 μg/ml) were incubated with heparin (average molecular weight: 17–19 kDa, Sigma) in PBS with 1 mM EDTA, 1 mM AEBSF and 1 mM TCEP at 37 °C for 72 h, as described [31]. The molar ratio of Tau:heparin was approximately 4:1. The kinetics of Tau assembly were monitored by the fluorescence of Thioflavin T (ThT), as described [10, 15, 49].

### Electron microscopy

For a semi-quantitative assessment of Tau filaments, negative-stain electron microscopy was used, as described [12]. Briefly, aliquots of heparin-induced assembled recombinant Tau were negatively stained with 2% uranyl acetate and observed using a Tecnai G2 Spirit TEM. Images were taken at × 6500 magnification.

### Stereology

Following a pilot study using the optical fractionator probe of StereoInvestigator 11 (MBF Bioscience), we opted for a total of eight sections/animal spanning L3-L5 of the spinal cord, in a random manner from a total of 96 sections (using 1:12). Section thickness was determined using StereoInvestigator 11 software. For each section, the outline of the region of interest was traced under a × 5 objective starting from the middle of the central canal and contouring the grey matter of the ventral horn. The average area of the delineated region was 351,498 ± 3983 μm^2^ for the P301S Tau group and 361,536 ± 7497 μm^2^ for the wild-type group (the difference between the two groups was not significant). For the human P301S Tau x mouse Tau knockout line, the average area sampled was 308,041 ± 13,436 μm^2^; it was 326,447 ± 17,847 μm^2^ for the age-matched human P301S Tau mice (the difference between the two groups was not significant). By using the optical fractionator probe (grid size 65 × 65 μm^2^; height 22 μm; guard height 3 μm; counting frame 50 × 50 μm^2^), NeuN-positive cells with a diameter of at least 30 μm and their nuclei within the dissector volume were counted using the × 100 objective, and the number of motor neurons per lumbar spinal cord calculated. The investigator was blinded with respect to the nature of the groups. Statistical analyses were performed using a one-way ANOVA test, followed by Tukey’s multiple comparisons test (Prism, GraphPad Software, Inc.).

### Image quantitation

Photographs were taken using an Olympus BX41 microscope equipped with a Nikon Digital Sight DS-2Mv digital camera under a × 20 objective. Each picture amounted to 1600 × 1200 pixels. AT100 immunoreactivity was quantitated using the green channel of ImageJ (NIH). The threshold was set to: 134 in greyscale, and the circularity to: 0-infinity.

## Results

### AT100 immunoreactivity and motor neuron loss in mice transgenic for human P301S tau

Sections from the lumbar spinal cord of full-length human 0N4R P301S Tau transgenic mice were stained with antibody AT100 to identify Tau aggregates and anti-NeuN to detect motor neurons. The same was done for age-matched wild-type mice.

**Fig. 1:**
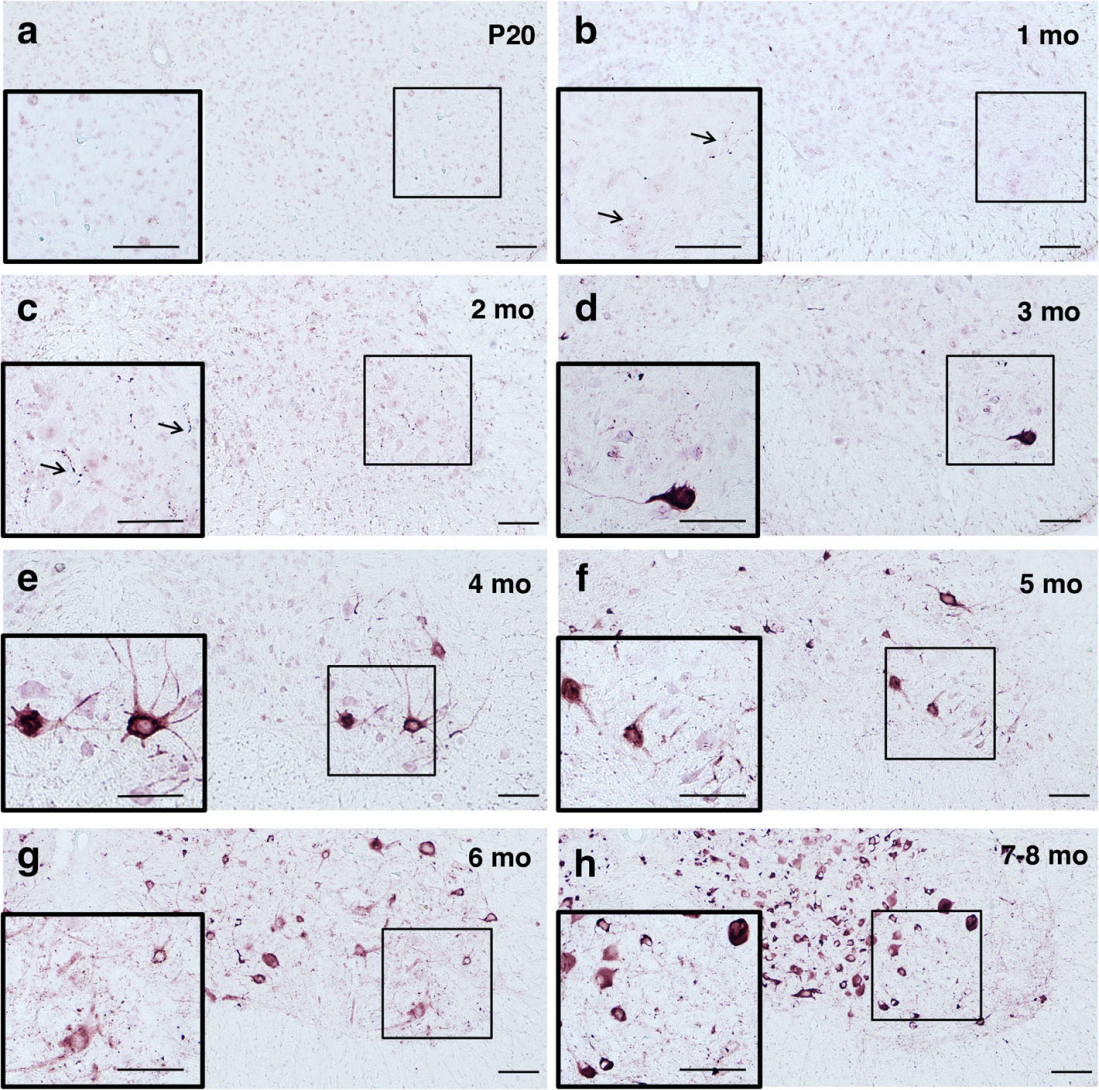
AT100 immunoreactivity in ventral horn of the lumbar spinal cord of mice transgenic for full-length human P301S Tau as a function of time. Insets depict the framed areas at higher magnification. Note the absence of AT100 immunoreactivity in (**a**) and the presence of stained dot-like structures in (**b**) and (**c**) (arrowed), prior to cell body staining (**d**-**h**). Scale bars, 100 μm

**Fig. 2:**
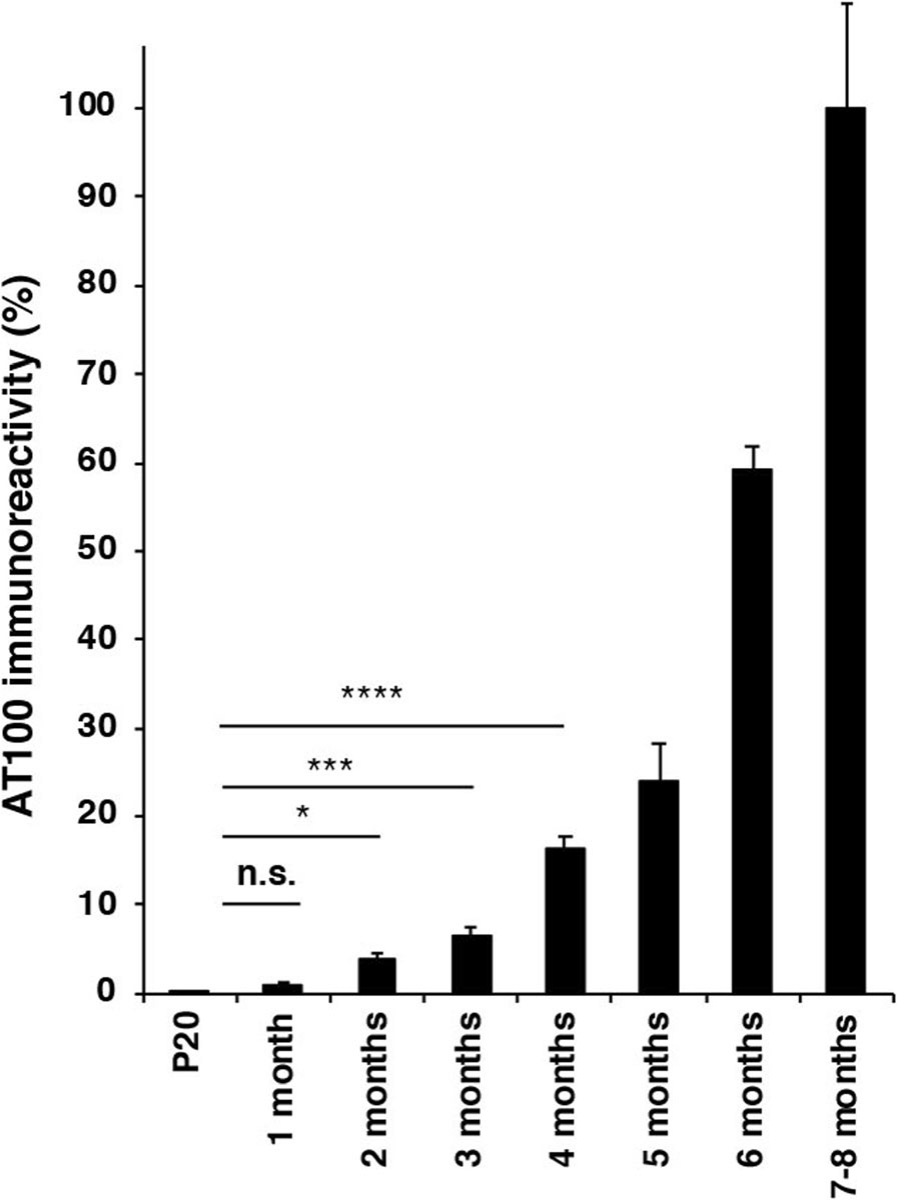
Quantitation of AT100 immunoreactivity in ventral horn of the lumbar spinal cord of mice transgenic for full-length human P301S Tau as a function of time (5 animals/group). The values at 7–8 months (end stage) are taken as 100%. One-way ANOVA [F (4,20) = 66.17, *p* < 0.0001], followed by Tukey’s multiple comparison test between P20 mice and those aged 1, 2, 3 or 4 months; n.s. = not significant; *p < 0.05; ***p < 0.001; ****p < 0.0001. The results are expressed as means ± S.E.M

Figs. 1 and 2 show the time course of AT100 immunostaining qualitatively and quantitatively, respectively. There was no staining in wild-type and 20-day-old (P20) transgenic mice, indicating the absence of Tau aggregates. However, in 1-month-old transgenic mice, a small number of AT100-positive, dot-like structures, was present in neurites throughout the lumbar spinal cord. A similar picture was observed at 2 months, but the number of dot-like structures had increased (*p* < 0.05 relative to P20, using 1-way ANOVA). Prominent cell body staining for AT100 was observed in addition to increased neuritic staining in transgenic mice aged 3 months (*p* < 0.001 relative to P20). The number of immunoreactive nerve cell bodies and terminals steadily increased between 3 months and the end stage at 7–8 months of age.

**Fig. 3:**
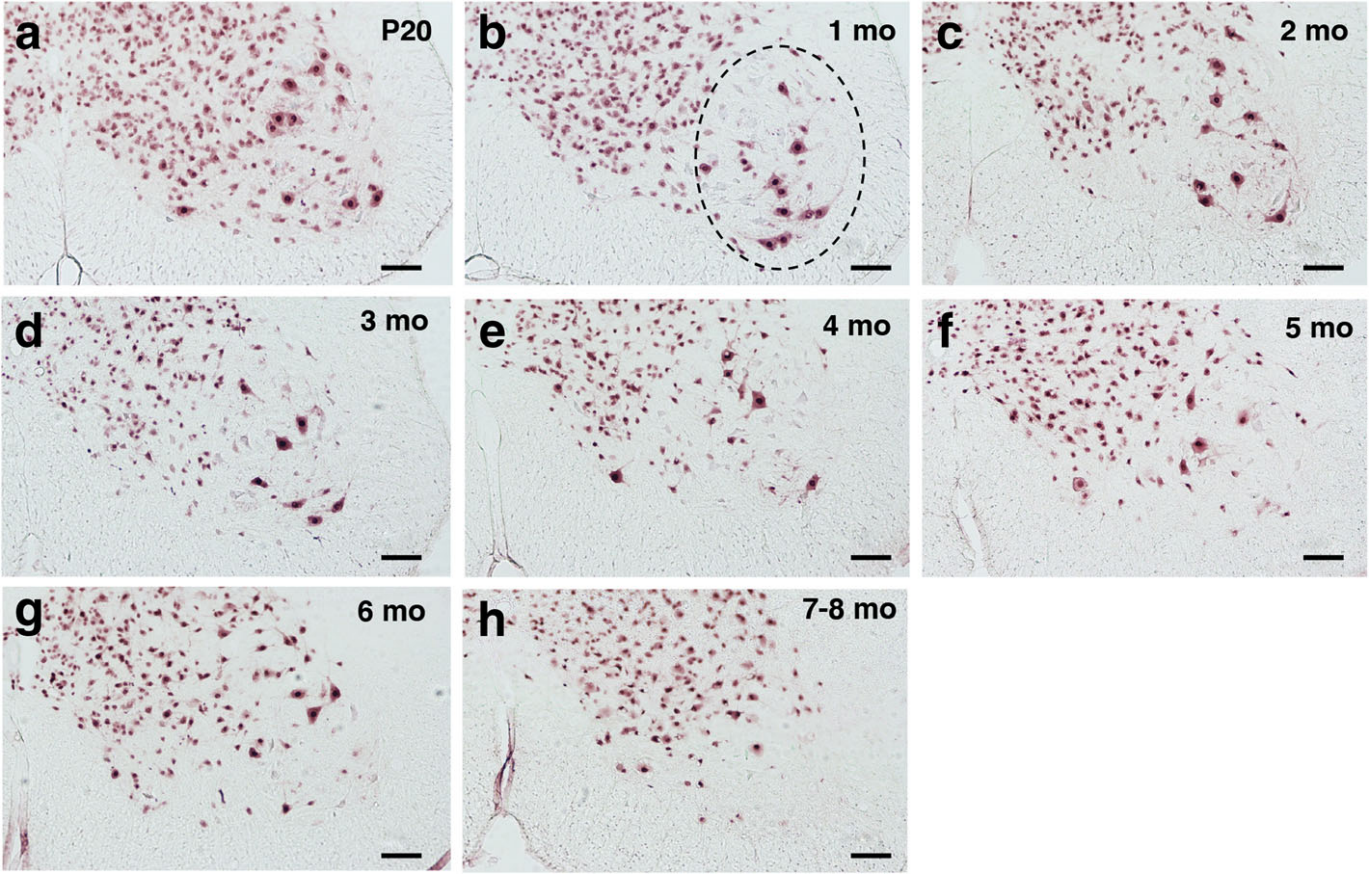
NeuN immunoreactivity in ventral horn of the lumbar spinal cord of mice transgenic for full-length human P301S Tau as a function of time. Motor neurons are large cells in the ventral horn (circled with a dashed line in **b**). Scale bars, 100 μm

**Fig. 4:**
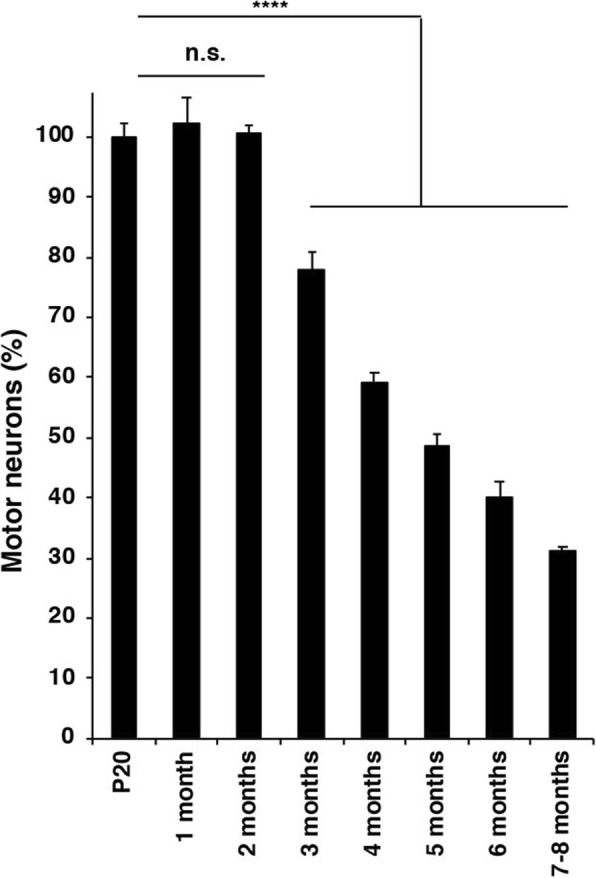
Quantitation of motor neuron numbers in ventral horn of the lumbar spinal cord of mice transgenic for full-length human P301S Tau as a function of time (5 animals/group). The values at P20 are taken as 100%. One-way ANOVA [F (7,32) = 152.1, *p* < 0.0001], followed by Dunnett’s multiple comparison test; n.s. = not significant; ****p < 0.0001. The results are expressed as means ± S.E.M

Figs. 3 and 4 show the time course of NeuN staining. The number of motor neurons in transgenic P301S Tau mice aged 20 days, 1 month and 2 months did not differ significantly from one another or from the number of motor neurons in wild-type mice. In transgenic mice, significant motor neuron loss (22% reduction relative to P20) was first observed at 3 months of age. It reached 41% at 4 months, 51% at 5 months, 60% at 6 months and 69% at 7–8 months. A Pearson product-moment correlation coefficient was computed to assess the nature of the relationship between AT100 immunoreactivity and number of motor neurons. A significant negative correlation was seen, r = − 0.85, *n* = 5, *p* = 0.007.

### The relevance of murine tau in mice transgenic for human P301S tau

**Fig. 5:**
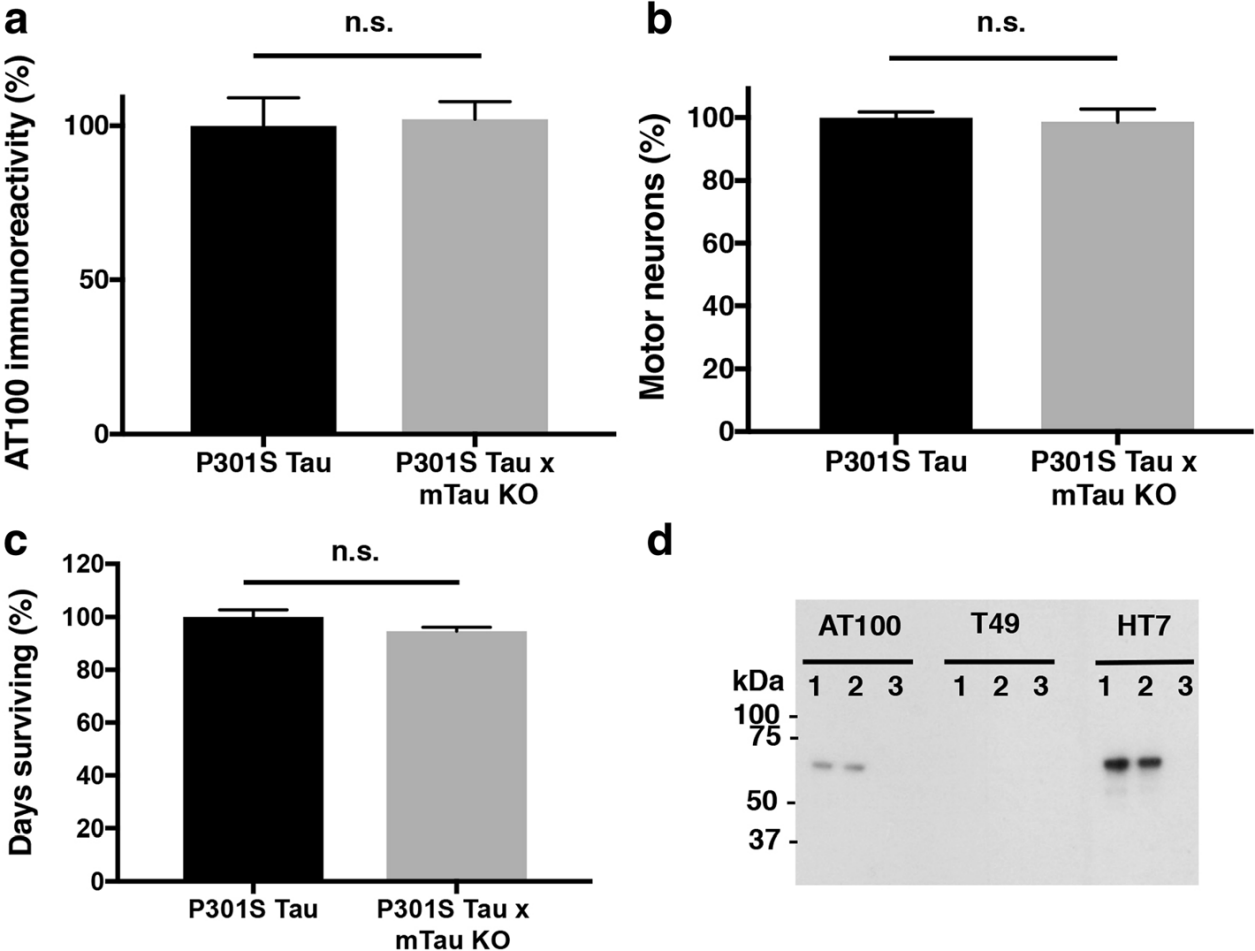
Relevance of murine Tau for assembly and neurodegeneration. The values of AT100 immunoreactivity (**a**) and motor neuron numbers (**b**) of homozygous 7–8-month-old mice transgenic for full-length human P301S Tau on a normal murine Tau background are taken as 100%. The results are expressed as means ± S.E.M. (5 animals/group). Two-tail unpaired student t-test revealed no significant difference (*p* = 0.78 and *p* = 0.85 respectively). **c** Survival of mice transgenic for full-length human P301S Tau compared to those on a murine Tau null background (20 animals/group). Two-tail unpaired student t-test revealed no significant difference (*p* = 0.09). n.s., not significant. **d** Immunoblots of sarkosyl-insoluble Tau from spinal cord of 7–8-month-old mice transgenic for full-length human P301S Tau, in presence (lane 1) or absence (lane 2) of murine Tau. Lane 3 shows lack of immunoreactivity in an age-matched wild-type mouse. Antibody AT100 is specific for Tau phosphorylated at T212/S214/T217 and identifies filamentous Tau. T49 recognises murine Tau, whereas HT7 is specific for human Tau

The contribution of murine Tau to aggregation and loss of motor neurons was investigated by comparing the lumbar spinal cords of end stage human P301S Tau mice and human P301S Tau x mouse Tau knockout mice (P301S Tau x mTau KO). Quantitation of AT100 immunoreactivity showed no significant differences between the two groups (Fig. 5a). The same was true when motor neuron numbers were counted using unbiased stereology (Fig. 5b). Additionally, the average survival times of human P301S Tau mice were similar to those of human P301S Tau x mTau KO mice (*n* = 20) (Fig. 5c). Lastly, upon extracting Tau filaments from end stage P301S Tau and P301S Tau x mTau KO spinal cords, and running these extracts by immunoblotting, both lines showed similar levels of filamentous Tau (as judged by AT100 immunoreactivity). Aggregates were made of human mutant Tau, since no murine Tau was detected in the aggregates, as judged by the lack of T49 immunoreactivity (Fig. 5d).

### Filament assembly of human P301S tau is required for neurodegeneration in transgenic mice

**Fig. 6:**
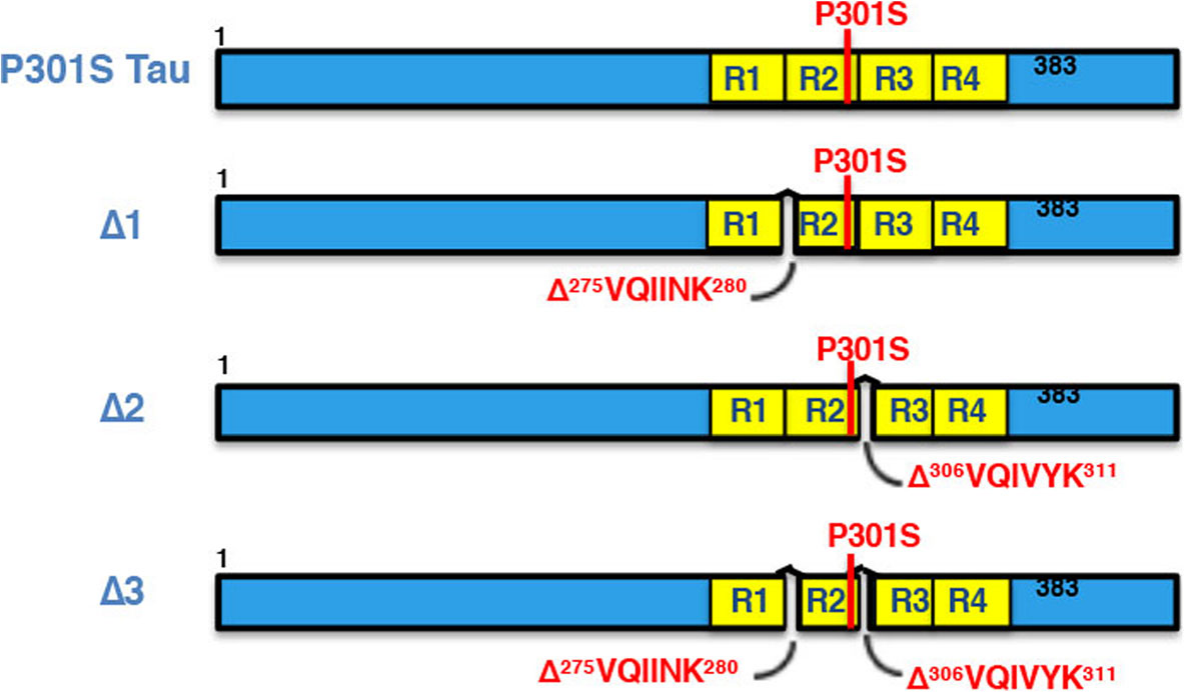
Schematic of constructs used to generate transgenic lines and recombinant Tau. Four variants were made: 1) Full-length human 0N4R Tau with the P301S mutation (P301S Tau), 2) 0N4R P301S Tau with deletion of ^275^VQIINK^280^ (Δ1), 3) 0N4R P301S Tau with deletion of ^306^VQIVYK^311^ (Δ2), and 4) 0N4R P301S Tau with deletions of ^275^VQIINK^280^ and ^306^VQIVYK^311^ (Δ3)

**Fig. 7:**
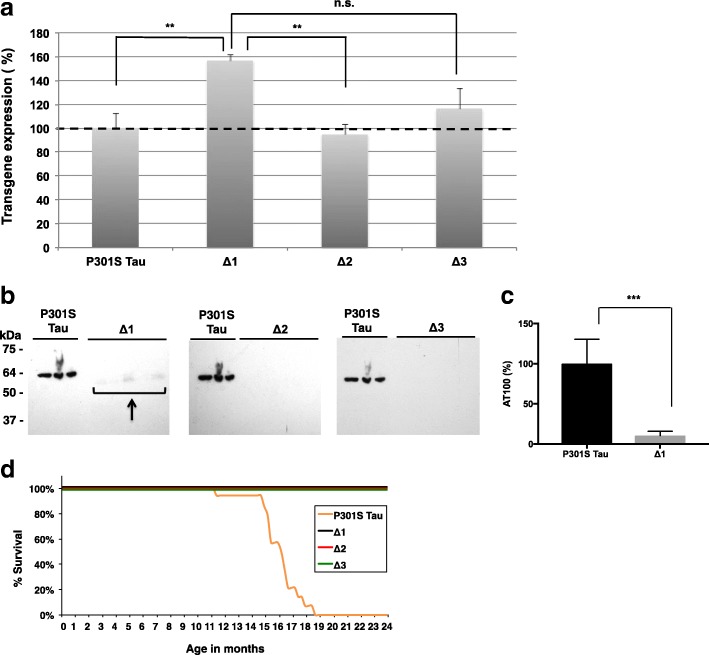
Mice transgenic for full-length human P301S Tau and for Δ1, Δ2, Δ3. **a** Relative levels of transgene expression, as measured by ELISA, with those of full-length human P301S Tau taken as 100%. One-way ANOVA [F (3,16) = 6.109, *p* = 0.0057], followed by Dunnett’s multiple comparison test (**b)** Sarkosyl-insoluble Tau in the brains of mice transgenic for human P301S Tau (16 months) and transgenic for Δ1, Δ2 and Δ3 (24 months) immunoblotted for AT100. The n numbers were as follows: 3 for P301S Tau, 5 for Δ1, 5 for Δ2, and 5 for Δ3. The arrow points to low levels of sarkosyl-insoluble Tau in line Δ1. **c** Quantitation of the sarkosyl-insoluble samples run by Western Blot, with the values for P301S Tau taken as 100%. One-way ANOVA [F (3,14) = 18.19, *p* < 0.0001], followed by Tukey’s multiple comparisons test. The results are expressed as means ± SEM. *** *p* < 0.001, n.s. = not significant. **d** Survival plot of the human P301S Tau transgenic line compared to lines Δ1, Δ2, and Δ3

To examine the relationship between β-sheet-dependent filament assembly of Tau and neurodegeneration, we generated mice transgenic for P301S Tau with deletion of residues ^275^VQIINK^280^ (line Δ1), ^306^VQIVYK^311^ (line Δ2), or ^275^VQIINK^280^ and ^306^VQIVYK^311^ (line Δ3), as shown in Fig. 6. After assessing the expression levels of human mutant Tau in lines Δ1-Δ3 relative to those in the heterozygous full-length P301S Tau transgenic line by ELISA (Fig. 7a), the presence of sarkosyl-insoluble Tau in brain was investigated. While abundant sarkosyl-insoluble Tau was present in the full-length P301S Tau transgenic line at 16 months, none was seen in the Δ2 and Δ3 lines at 24 months of age. A small amount of sarkosyl-insoluble Tau was present in mice from line Δ1 (Fig. 7b, c) at 24 months, which showed an uncoordinated gait at this age, characteristic of the early stages of phenotype. Heterozygous P301S Tau mice usually reach end stage 3 to 4 months following onset of phenotype. Mice from the full-length P301S Tau line suffered a severe paraparesis and reached end stage at around 16–19 months of age, whereas mice from lines Δ2 and Δ3 were fully mobile at 24 months and had a normal lifespan (Fig. 7d).

### Human P301S tau assembly in vitro requires β-sheet structure

**Fig. 8:**
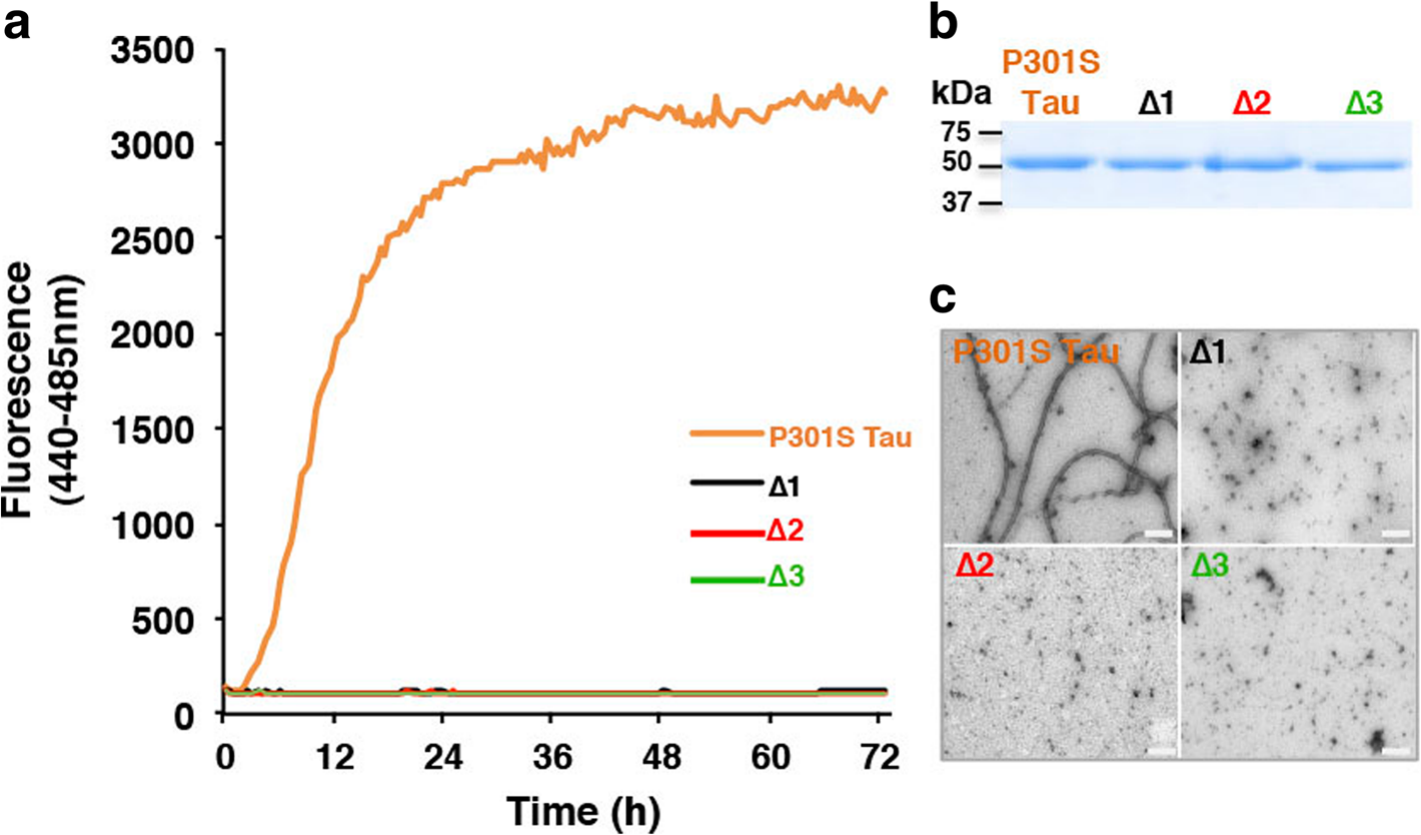
Heparin-induced assembly of recombinant Tau. **a** Heparin-induced assembly of recombinant Tau, monitored over time using Thioflavin T fluorescence. P301S Tau is shown in orange, Δ1 in black, Δ2 in red and Δ3 in green. **b** Coomassie-stained gel of purified proteins. **c** Negative stain electron microscopy of assembled recombinant Tau. Note that full-length recombinant P301S Tau assembled into filaments, but that Δ1, Δ2, and Δ3 proteins did not form filaments. Scale bars, 200 nm

Unlike recombinant 0N4R P301S Tau, 0N4R P301S Tau lacking residues ^275^VQIINK^280^ (Δ1), ^306^VQIVYK^311^ (Δ2) or both hexapeptides (Δ3) failed to form β-sheet structure (Fig. 8a) or filaments (Fig. 8c) following incubation with heparin.

## Discussion

Neurodegeneration and propagation of pathology are hallmarks of human tauopathies [16]. Mice transgenic for human mutant P301S Tau exhibit major features of the human diseases, including abundant filamentous Tau inclusions and nerve cell loss [1]. Because of its consistency, this mouse line has been found to be suitable for drug discovery studies [47]. However, the relationship between Tau aggregation and nerve cell loss is unknown. Here we show an inverse correlation between AT100 immunoreactivity and the number of motor neurons in the lumbar spinal cord of mice transgenic for human P301S Tau.

We showed previously that AT100 detects filamentous human Tau in transgenic mice [1, 6, 7]. It remains to be seen if it also detects other forms of Tau aggregates, such as oligomers. Although AT100 is a phosphorylation-dependent anti-Tau antibody (pT212/pS214/pT217), it only labelled insoluble non-sonicated Tau in mice transgenic for human P301S Tau [1, 7]. By immunohistochemistry, AT100 detected predominantly dot-like, neuritic inclusions in human P301S Tau transgenic mice aged 1–2 months. These findings suggest that Tau assemblies form in nerve terminals innervating muscle, before they appear in nerve cell bodies, as reported in another line of mice transgenic for human P301S Tau [48]. From 3 months onwards, the number of Tau aggregates increased in both terminals and cell bodies. End stage P301S Tau mice have neurogenic muscle atrophy [1]. P301S Tau transgenic mice and AT100 immunoreactivity have been instrumental for the experimental demonstration of the prion-like properties of aggregated Tau [6] and the finding that short Tau filaments have the greatest seeding activity [22]. Previous experiments in a mouse model of Tauopathy reported seeding activity before detectable neuropathology or accumulation of insoluble protein [20]. A study using a different mouse line transgenic for human P301S Tau reported the presence of Tau seeds in the brain at 3–5 weeks of age [47]. This agrees with the presence of AT100-immunoreactive structures in the spinal cord at 4 weeks reported here. It remains to be seen when Tau seeds can first be detected in the lumbar spinal cord of our mice transgenic for human P301S Tau.

Unlike AT100-immunoreactive structures, which were present from 1 month onwards, significant motor neuron loss was only present at 3 months of age. Thus, intraneuronal build-up of Tau aggregates may have been necessary for nerve cell loss. Recent studies report that Tau can disrupt nucleocytoplasmic transport [9, 33]. However, we did not observe any obvious disruption of nucleocytoplasmic transport or change in nuclear morphology in mice transgenic for human mutant P301S Tau. Motor neuron loss increased in parallel with the accumulation of AT100 immunoreactivity, with a 69% reduction at end stage. A correlation between AT100 immunoreactivity and motor neuron loss was observed, suggesting a causal connection. Secondary nucleation processes may also have played a role [24, 42].

The relevance of murine Tau for end stage AT100 immunoreactivity, motor neuron loss, and survival was investigated using human P301S Tau transgenic mice that had been crossed with a mouse Tau knockout line. The presence of murine Tau played no significant part in the above, consistent with Tau inclusions being made almost exclusively of mutant human Tau [1] and the P301S mutation increasing the aggregation propensity of Tau [17, 26]. In humans with frontotemporal dementia caused by the P301L mutation in *MAPT*, only protein produced from the mutant allele was found in Tau inclusions [29]. Moreover, aggregates of P301L Tau could seed the aggregation of P301L Tau, but not of wild-type Tau [2].

To further analyse the relationship between Tau aggregation and neurodegeneration, mice transgenic for human P301S Tau that lacked residues 275–280, residues 306–311 and both hexapeptides were generated and analysed. These sequences are necessary for heparin-induced filament formation of wild-type Tau in vitro [25, 45, 46]. This is, to our knowledge, the first report detailing the in vivo effects of such hexapeptide deletions in a transgenic model expressing full-length human mutant Tau. Having quantitated the levels of expression of human P301S Tau in the different transgenic lines, we observed a small amount of sarkosyl-insoluble tau in the brains of mice transgenic for Δ1 at 24 months of age. This was in agreement with an in vitro study showing that residues ^306^VQIVYK^311^, but not ^275^VQIINK^280^, were necessary for heparin-induced assembly of wild-type K18, the 4 repeat domain of human Tau (residues 244–372), into filaments [25]. One might therefore expect a longer incubation time of Δ1 with heparin to eventually yield a small number of Tau filaments. This would not be the case for Δ2 and Δ3. These findings are reminiscent of *Drosophila* lines expressing human wild-type Tau (0N4R) lacking residues 306–311 that developed no detectable neurodegeneration and significantly less hyperphosphorylated Tau than fly lines expressing full-length Tau [34]. We failed to observe significant levels of sarkosyl-insoluble Tau in mouse lines Δ2 and Δ3 at 24 months of age. As described before, mice transgenic for full-length P301S Tau developed abundant Tau filaments, nerve cell loss and a severe paraparesis at 16–19 months of age. None of the Δ1-Δ3 lines developed motor impairment.

High-resolution structures of the cores of Tau filaments assembled from wild-type recombinant 4R Tau and heparin have been shown to be polymorphic [51]. The most common structure extends from residues 272–330 of Tau and encompasses residues 275–280 and 306–311. P301 is located in the partially disordered hammerhead arc. Since proline residues interrupt hydrogen bond interactions across filament rungs, replacing P301 with L or S may facilitate filament formation by stabilising local structure. Recombinant Tau mutated at residue 301 (P to L or S) forms significantly more heparin-induced filaments than wild-type protein [17].

Unlike human P301S Tau, the expression of one isoform of wild-type human Tau in transgenic mice does not lead to filament formation or neurodegeneration. We show here that deletion of residues ^275^VQIINK^280^ and ^306^VQIVYK^311^ prevents the assembly of human P301S Tau in transgenic mice. Similar findings have been reported in a cell model of seeded Tau aggregation [10]. Interestingly, deletion of amino acid 280 (ΔK280) results in a significantly greater propensity of Tau to assemble into filaments [3, 36]. This deletion causes frontotemporal dementia in humans, but probably through a mechanism involving mRNA splicing [44]. It thus appears that the ΔK280 mutation increases filament assembly of recombinant Tau, whereas its deletion in the absence of residues ^275^VQIIN^279^ abolishes filament assembly. However in vivo, expression of full-length ΔK280 Tau did not yield Tau filaments or overt neurodegeneration [8]. Our findings are reminiscent of those of Mocanu [30], in which mice transgenic for the K18 Tau fragment with ΔK280 showed Tau filaments and nerve cell loss. Since most in vitro studies of Tau assembly were carried out in the presence of heparin, and since monomeric Tau is very soluble, other cofactors and/or post-translational modifications may be required for the assembly of human P301S Tau in brain [12, 13, 32]. It will be interesting to determine high-resolution structures of wild-type and mutant 4R Tau filaments.

Taken together, the present findings establish a close correlation between Tau assembly and neurodegeneration in mice transgenic for human mutant P301S Tau.
